# Editorial: Insights in sports social science

**DOI:** 10.3389/fspor.2023.1219674

**Published:** 2023-05-26

**Authors:** Hans Westerbeek, Gayle McPherson, Jess C. Dixon

**Affiliations:** ^1^ Institute for Health and Sport, Victoria University, Melbourne, VI, Australia; ^2^ School of Business and Creative Industries, University of the West of Scotland, Paisley, United Kingdom; ^3^ Faculty of Human Kinetics, University of Windsor, Windsor, ON, Canada

**Keywords:** sport business insights, social science, innovation, future, sport management and marketing

**Editorial on the Research Topic** Insights in sports social science

We are now entering the third decade of the 21st Century, and, especially in the last years, the achievements made by natural and social scientists have been exceptional, leading to major advancements in the fast-growing field of Sports and Active Living. This collection of articles is part of a series of Research Topics across the field of Sports and Active Living. This multi-disciplinary, editorial initiative is focused on new insights, novel developments, current challenges, latest discoveries, recent advances, and future perspectives in the field of sports social science. The goal of this special edition Research Topic was to shed light on the progress made in the past decade in the sport social science field, and on its future challenges to provide a thorough overview of the field. This article collection that has contributions from Canada, throughout Europe, UK, USA and South Africa will inspire, inform and provide direction and guidance to researchers in the field. This collection considers the findings from 11 research teams that from a variety of perspectives have identified current challenges in several sub-disciplines, and who have applied different methodologies to address those challenges. The different viewpoints are reflected in the types of articles that were included in the Research Topic, including articles containing original research, perspectives, a brief research report, a conceptual analysis, and a systematic review. What follows is a brief outline of the various projects.

Testa conducted a study into extremism in the Bosnia and Herzegovina (BiH) football terraces, focusing on risk factors that govern the “entry” of BiH youth into extreme hard-core football fans groups and prolong their involvement in them. The study provided recommendations for BiH policymakers, security agencies, and football federations and clubs to understand and effectively respond to this threat for public security in BiH.

Partly in response to the global Covid-19 pandemic, Weese et al. proposed transformative changes in what sport management academicians teach, how they teach, and where they teach, to facilitate working in flexible environments and across areas. Sport management professors are offered suggestions to help them seize the opportunities arising from the changing sports landscape and emerging entrepreneurial ventures.

In a paper focusing on participants in the Danish version of the reality TV-show Alone in the Wilderness (AIW), Andkjaer and Ishoi explored their motives, values, and experiences of being part of the show. The study used a hermeneutic approach, and the analysis was based on a 6-phased thematic analysis. The findings suggest that the motives and values of the participants reflect ideas that may be related to the solo experience and the Nordic tradition of friluftsliv (simple life in the outdoors). The study presents new empirically based knowledge on the motives, values, and experiences of people participating in AIW and how these can be understood as part of outdoor education and recreation and as a cultural phenomenon in late modern society.

In the USA, the National Football League (NFL) and its teams face challenges with diversity, equity, and inclusion (DEI). In their perspective piece, DeMartini and Butler investigated NFL teams' utilization of organisation employees dedicated to DEI, utilizing a content analysis of publicly available data. Their findings conclude that NFL teams lag behind other American businesses in their adoption of Chief Diversity Officer (CDO) roles. Only 31.25% of NFL teams had a dedicated DEI staff person. Three additional teams host diversity councils utilizing employees with other job responsibilities. The study suggests that to address these challenges and move forward, NFL teams should create CDO roles with appropriate reporting relationships, well-crafted position responsibilities, generous resources, and qualified and experienced employees.

Seibeth et al. explored stories of national identity development from the perspective of youth football players with Turkish background in German youth elite football. The study used 10 expert interviews and biographical mappings to identify specific types, strands, and trajectories of national identity development. The findings illustrate three types of narratives on national identity development: “going with the nomination(s),” “reconsidering national belonging,” and “adding up chances.” The study concludes that national identity development in youth elite sport is a complex process.

In a paper that asked the question of how arena-anchored urban development projects fit into a local city's tourism economy, Barry et al. positioned professional sports teams as the anchor tenants of sport facilities to generate development in the city. The study draws data from two cities, Columbus, Ohio, and Detroit, Michigan, using interviews with leaders and content analysis. Their results indicate that growing the visitor economy through arena anchored urban development relies on planned placemaking *via* the strategic approach of bundling diverse amenities together. These findings provide valuable feedback to those cities considering arena development projects, and how the arenas may be combined with other civic amenities to undergird the local visitor economy.

In their conceptual analysis, Zheng and Mason argued that the emergence and proliferation of new media technologies have drastically changed the media landscape. This has created a much more complicated cross-media environment that unites popularity and personalization, structure and agency. This changing environment creates industry transformations, and adapting to these transformations will lead to the accelerated and ongoing evolution of the professional sport industry and its success in the digital media age.

Emerging economies are increasingly hosting large-scale and mega sport events as they are viewed as key factors in local and national development strategies. Knott and Tinaz argued that a variety of legacies have predominated the literature over the past two decades. However, it is proposed that there is a difference in the types of legacies anticipated or realised within emerging economies. Therefore, this systematic review aimed to determine the types of legacies anticipated or realised by emerging economies as a result of hosting sport events, and to determine if these differ from those of more economically developed nations. The study confirms legacy as a growing body of knowledge in emerging nations, aligned with increasing event hosting. A conceptualisation of key legacy areas for emerging nations is proposed, including social development; politics, soft-power and sport-for-peace; the economics of tourism, image and branding; infrastructure and urban development; and sport development.

The environmental impacts of shadow stadia, which are the facilities left behind after new stadium development, are not fully understood. Limited research exists on how the immediate neighbourhood anchored by pre-existing venues cope in the shadows of these new development plans and the loss of a sport venue and its events. In their perspective article, Barry et al. discuss current advances in the academic literature on the circular economy. They present a comprehensive categorisation of shadow stadia globally and future opportunities on integrating circularity into best practices. By doing so, this perspective article highlights several areas of future investigation that should be considered and planned for when major league sports teams and city leaders move their team and build new facilities.

Sport marketing research has much to gain from engaging with critical social science assumptions, worldviews, and perspectives to examine complex issues in sport. Evans et al. argued that, historically, sport marketing research has adapted traditional research approaches from the parent marketing discipline to sport. This paper offers two research propositions, each accompanied by four actional recommendations, to advance the field of sport marketing in meaningful and impactful ways. The paper employs a particular focus on the marketing campaigns that activate and promote corporate partnerships in sport to frame the two propositions, which discuss consumer culture theory and the circuit of culture as two important frameworks that begin building bridges between critical social science and sport marketing research.

In the final article of the Research Topic, Pereira et al. discussed the importance of nautical tourism as a potential product to promote and develop tourist destinations in Europe. The study focuses on analysing the strategic alliances established by nautical stations in Portugal for the development of nautical tourism products, including their strategic goals and sustainable environmental practices. A content analysis of 17 Portuguese nautical stations' application forms collected between September and December 2021 showed that strategic alliances between nautical stations had multiple strategic objectives, including structuring the tourism offer, increasing governance, and promoting and marketing nautical tourism. The study concluded that future scientific research is needed to operationalize the objectives underlying the formation of strategic alliances and the environmental practices developed by nautical stations.

